# Health worker experience of heat-related discomfort, service delivery, and related coping and adaptation in Ghana

**DOI:** 10.1186/s12982-026-02537-2

**Published:** 2026-08-25

**Authors:** Delali Benjamin K. Dovie, Lois Antwi-Boadi, Abena Fosuaa Ofosu Koranteng, Wilhemina Quaye, Margaret Appiah

**Affiliations:** 1 Regional Institute for Population Studies, University of Ghana, Legon, Ghana; 2 Science and Technology Policy Research Institute, Council for Scientific and Industrial Research, Accra, Ghana; 3 Department of Environment and Public Health, University of Environment and Sustainable Development, Somanya, Ghana

**Keywords:** Cognitive function, Health system, Infrastructure, Indicators, Climate adaptation, Rising temperatures, Ghana

## Abstract

**Introduction:**

Mean daily temperatures are rising and may affect health system functioning, yet evidence remains limited on how health workers experience and respond to heat-related discomfort across interconnected health system domains. This study explored health workers’ experiences of how heat-related discomfort influences workforce functioning, service delivery, and health infrastructure, and related governance and adaptation within Ghana’s health system.

**Methods:**

Eighteen focus group discussions and fifty-six key informant interviews were conducted with health directors, facility managers, nurses, and public health officers across six regional clusters. The WHO Health System Building Blocks framework guided the thematic data analysis using MAXQDA, with inductive coding to identify emerging patterns.

**Results:**

Heat-related discomfort was associated with fatigue, dehydration, reduced concentration, and challenges in maintaining regular work attendance and routines. Poor ventilation and unreliable power supply were perceived to intensify indoor heat, constrain usable workspace, and create challenges for medicine and vaccine storage. Effects of heat on service delivery included congestion, staff absenteeism, shortened consultations, and irregular patient attendance. Although formal governance and financing mechanisms for heat preparedness were perceived as limited, health workers described a range of behavioural, operational, and facility-level responses, including adjusting work schedules, triaging patients, reorganising workspaces, and planting shade trees.

**Conclusion:**

Participants’ experiences of strengthening heat preparedness may benefit from integrating heat management into occupational health regulations, improving facility design and ventilation, and providing resources for climate-related adaptation. Such measures could help support the continuity and resilience of health service delivery under rising temperatures.

**Supplementary Information:**

The online version contains supplementary material available at 10.1186/s12982-026-02537-2.

## Introduction

Rising mean daily temperatures globally are increasingly reported to pose significant risks to worker comfort, safety, and productivity including the health workforce. In sub-Saharan Africa (SSA), where baseline temperatures are high and cooling infrastructure is limited [1, 2], heat-related discomfort has become an important occupational public health concern [3, 4]. Whilst the focus of the scientific community has largely been outdoor occupations such as in agriculture, construction, and mining [5–8], much less is known about health workers’ experiences in sustaining essential services and population wellbeing in resource-constrained environments [9, 10].

Thermal comfort from 18 to 24 °C (64–75 °F) supports optimal physical and cognitive functioning [11–15], but many facilities in SSA lack adequate ventilation, air conditioning, or reliable power supply [16–18]. As a result, indoor conditions often mirror outdoor heat attributes in contributing to fatigue, reduced worker concentration, slower work pace, and increased absenteeism [12–15, 19], thus negatively impacting clinical care, where sustained attention, empathy, and infection control are less attended to [19, 20].

Despite the recognition that temperature affects occupational performance [11, 13, 14, 21], empirical evidence on how heat-related discomfort influences health system functioning remains limited amidst the lack of established measurable metrics. Field studies demonstrate how heat-related discomfort is experienced and interpreted by health workers themselves, and how these experiences help explain effects on service delivery and health infrastructure [5, 19, 22]. However, empirical evidence remains sparse on health worker perceptions on the governance and financing enablers during heat-related discomfort, as well as how they cope and adapt under thermal stress. Notwithstanding, understanding these relationships is crucial for designing climate-resilient health systems planning. Hence, guided by the World Health Organisation (WHO) Health System Building Blocks [23], this study assesses the impacts of health workforce exposure to heat and related discomfort on their functionality and wellbeing. It also evaluates the potential consequences for service delivery and infrastructure resilience, analyses governance and financing mechanisms that shape response capacity, and identifies adaptation strategies across individual, facility, and health system levels. The study was framed within the proposition that exposure to heat reduces health worker performance and wellbeing and disrupts service delivery and infrastructure functionality, yet effective governance and adequate financing structures may lessen these negative effects.

## Materials and methods

### Operational definitions

Heat-related discomfort: Conditions perceived by participants as uncomfortably warm or oppressive, which interfere with comfort, concentration, or functioning.

Coping: Short-term behavioural or operational adjustments made by individuals or facilities to manage immediate heat-related challenges and maintain basic functionality.

Adaptation: Sustained and purposeful actions or strategies at individual, facility, or system levels to minimise the negative effects of heat on health systems.

Health system: The institutions, resources, and organisations dedicated to the promotion, maintenance, or restoration of health. According to the WHO [23], a functional health system consists of: (i) governance and leadership, which ensure accountability and evidence-informed policy; (ii) health workforce (staff/health workers responsible for delivering effective health services); (iii) health information systems which support research and surveillance; (iv) access to essential medicines, medical products and technologies; (v) financing mechanisms; and (vi) service delivery for efficient and equitable provision of care. For this study, health information systems and access to essential medicines, medical products, and technologies were combined under health infrastructure.

### Study setting

This cross-sectional qualitative study was conducted in Ghana, a tropical West African country characterised by high temperatures and distinct wet and dry seasons. The country experiences mean annual temperatures between 26 °C and 30 °C, with recent decades showing a steady warming trend and increasing frequency of heat events [24]. Ghana’s decentralised health system operates across the sixteen administrative regions through a network of community-based compounds, health centres, district hospitals, and regional or teaching hospitals [25]. Facility infrastructure, energy reliability, and cooling capacity vary considerably between urban and rural settings, shaping each facility’s ability to manage heat-related discomfort. Study districts were selected across all regions to reflect the ecological and infrastructural delivery.

**Fig. 1:**
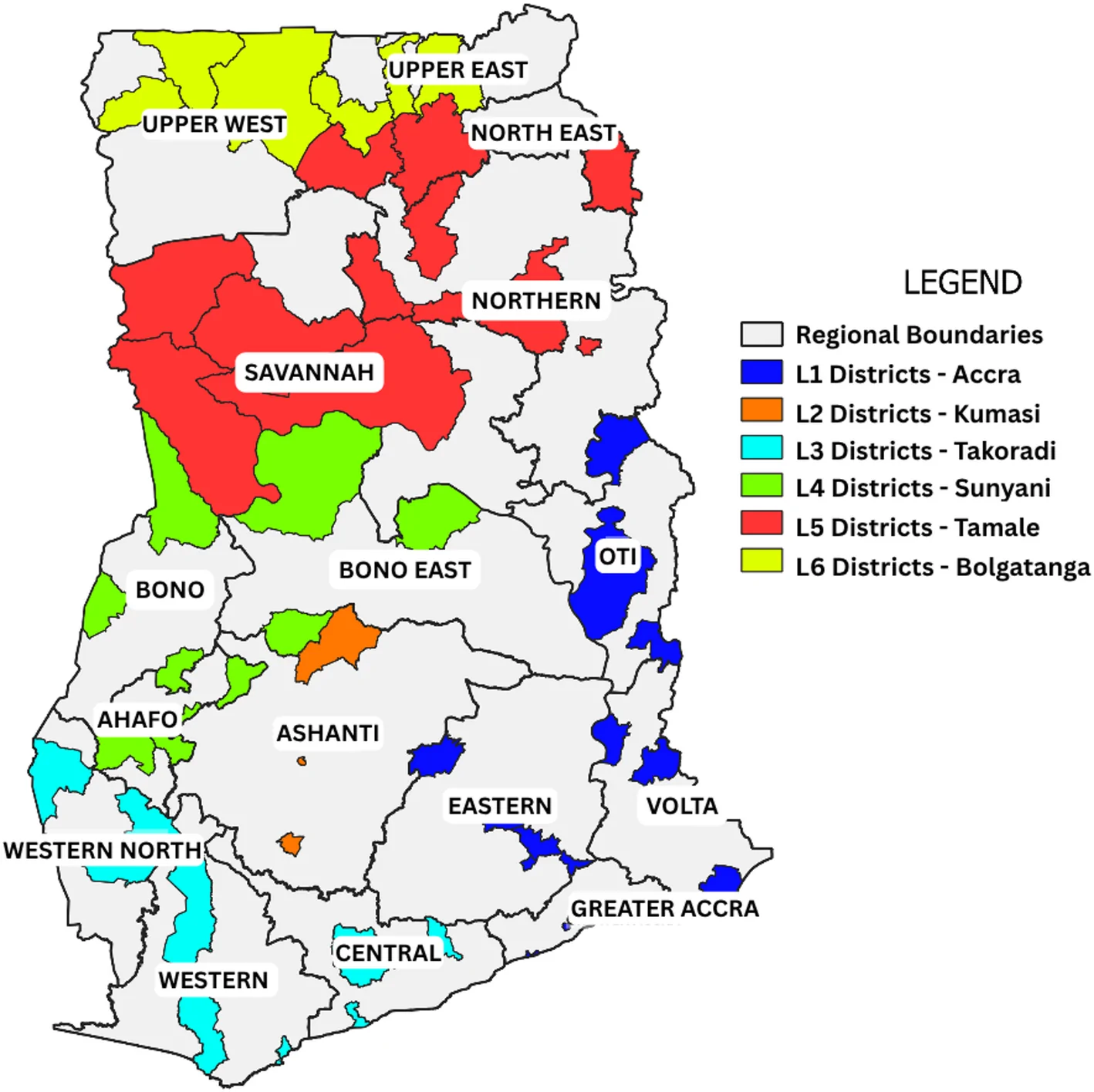
Map of Ghana showing selected study districts. Map created using QGIS 3.16 [26]. The regional and district boundary shapefiles were obtained from the Humanitarian Data Exchange (HDX) open data repository [27]

### Study design and participant recruitment

A qualitative exploratory design was used, as this approach is appropriate for topics with limited prior empirical attention [28, 29]. This enabled in-depth exploration of how heat-related discomfort affects the health workforce, service delivery, and infrastructure, as well as the coping and adaptation mechanisms. This study did not measure environmental temperature or physiological indicators of exposure to heat such as Wet Bulb Globe Temperature or heat stress index [30]. Instead, participants’ experiences and descriptions of heat-related discomfort were used to capture lived experiences in resource-limited facilities.

This study forms part of the Standards for Official Statistics on Climate-Health Interactions (SOSCHI) project, a four-year collaboration led by the Office for National Statistics (ONS) with partners including the Regional Institute for Population Studies (RIPS), the UK Health Security Agency (UKHSA), and the African Institute for Mathematical Sciences (AIMS) [31]. The broader methodology has been published elsewhere [32]. The present study which focuses on heat and health systems in Ghana aims to provide consistent, generalisable methods for capturing health risks attributable to climatic events, integrating real-world and local-level data, and supporting decision-making at national and global levels, using indicators. The WHO Health System Building Blocks framework guided both data collection and analysis, providing an organising structure for exploring system-level effects of heat-related discomfort.

Participants were purposively selected to ensure representation across Ghana’s administrative regions. Districts were grouped across six regional clusters (L1 to L6) (Fig. 1; Table 1). A total of 18 focus group discussions (FGDs) and 63 key informant interviews (KIIs) were conducted. The number and composition of FGDs and KIIs were determined analytically rather than by numerical targets. Sampling was structured to capture functionally distinct categories of the health workforce and facility-level portfolios most directly implicated in exposure to heat, service delivery, and facility operations. A pretesting phase informed the selection of cadres and roles whose routine responsibilities involved prolonged exposure to heat, decision-making under thermal stress, or oversight of facility operations. FGDs were therefore organised to capture shared experiential patterns within key workforce categories, while KIIs targeted managerial and supervisory portfolios with system-level responsibilities. Selection of participants was also guided by thematic saturation, with the final sample reached when no new concepts emerged within or across clusters.

Eligible participants had lived or worked in the facility location for at least three years and were familiar with local climatic and facility conditions. Participants included District/Municipal/Metropolitan Health Directors (HD), Public Health Officers and Nurses (PHO/N), Community-Based Health Planning and Services (CHPS) Coordinators (CHPS-C), Health Promotion Officers (HPO) and Health Information Officers (HIO), Principal Nursing Officers, and facility users (community members who access the services of the facility) [32]. Although facility users were included in the broader project to provide contextual insights on healthcare access, this paper focuses exclusively on health workers because the aim was to examine workforce, service, infrastructure, governance, and financing dimensions of heat-related discomfort. Therefore, only health workers were included in this analysis. No participants declined participation after being approached.

### Data collection

Data collection took place in 2025 by the expert members of the research team, with background in population health and experience conducting interviews in Ghanaian health settings. The interviewers were not part of participants’ organisational structures, minimising power imbalances, and with no prior personal contacts with the participants. Therefore, researcher positionality was actively considered throughout data collection, from reflexive field journals to documenting assumptions, emerging impressions, and decision-making. Regular team debriefings were held to discuss potential biases, enhancing reflexivity, and promoting consistency. Participants were informed about the purpose of the study, the research team’s roles, and the voluntary nature of participation and the right to withdraw at any time before providing consent.

FGDs (18) were used to explore collective views, while KIIs (56) captured individual experiences (Table 1). Structured interview and discussion guides, pre-tested for clarity, focused on experiences of heat-related discomfort, coping and adaptation strategies, and measures to improve climate-health system resilience. The thematic version of the guides used for the study is provided as Supplementary File 1. All interviews were conducted in English and audio-recorded with consent. Ethical approval was obtained from the Institutional Review Board of the Ghana Health Service (GHS) numbered GHS-ERC:005/04/25, and permission to access study sites was granted by relevant health directorates. Participants provided written informed consent after receiving information on study purpose, voluntary participation, and confidentiality. Interviews and discussions were held privately in health facilities and various meeting points during working hours to minimise service delivery disruption and ensure free expression.

**Table 1:** Distribution of FGDs and KIIs

Cluster (Code)	Regions Covered	FGDs	KIIs
L1 – Accra Cluster	Greater Accra, Eastern, Oti, Volta	3 (PHO/N, CHPS-C, HD)	18 (HPO; HIO; PNO; CHPS-C; PHO/N)
L2 – Kumasi Cluster	Ashanti	2 (PHO/N, CHPS-C, HD mixed)	4 (HIO; CHPS-C1; PHO/N)
L3 – Takoradi Cluster	Western, Western North, Central	4 (PHO separate; Public Health Nurses separate; CHPS-C; HD)	7 (DCO; PHO/N; CHPS-C; HPO)
L4 – Sunyani Cluster	Bono, Bono East, Ahafo	3 (PHO/N, CHPS-C, HD)	11 (HPO; HIO; PNO; PHO/N)
L5 – Tamale Cluster	Savannah, North East, Northern	3 (PHO/N, CHPS-C, HD)	13 (HPO; HIO; PNO; CHPS-C; PHO/N)
L6 – Bolgatanga Cluster	Upper East, Upper West	3 (PHO/N, CHPS-C, HD)	10 (HPO; HIO; PNO; CHPS-C; PHO/N)
Total	16	18 FGDs	56 KIIs

Source: Authors’ compilation from field data

Each FGD lasted about three hours, and each KII about one hour. Field notes captured contextual details, non-verbal observations, and researcher reflections, and were incorporated into analysis. Data collection and preliminary analysis occurred concurrently to guide follow-up questioning without repeated interviews. All recordings, transcripts, and field notes were securely stored under the custodianship of the Regional Institute for Population Studies (RIPS), University of Ghana, in line with institutional guidelines. Thematic saturation was assessed through ongoing review of coded transcripts and field summaries [28]. Coding logs in MAXQDA documented when redundancy was reached, providing an audit trail. Saturation was identified when interviews across different clusters yielded no new codes or themes, at which point data collection ended.

### Data analysis

Data were analysed thematically using MAXQDA 2024, and audio files transcribed verbatim and cross-checked for accuracy. Non-verbal cues were retained to preserve meaning. While deductive codes were derived from the WHO Health System Building Blocks and study objectives, inductive coding captured the emerging issues [33]. Coding was iterative and collaborative as three analysts refined a shared codebook, double-coded 15% of transcripts, and assessed intercoder agreement within MAXQDA. Any discrepancies were resolved through dialogue until consensus was achieved, towards enhancing dependability and analytic consistency. Transcripts were not returned to participants for further comment, correction, or feedback, rather higher level of validation of the data was carried out. A detailed coding tree reflecting deductive and inductive categories were established (Supplementary File 2).

Analytical rigour was also enhanced through reflexive memo-writing, peer-debriefing with two independent qualitative experts, and triangulation between FGDs and KIIs. These steps improved dependability and confirmability by documenting analytic decisions and ensuring findings reflected participants’ perspectives rather than researcher interpretation. Five overarching themes were identified: (i) health workforce, (ii) service delivery, (iii) infrastructure, (iv) governance and financing, and (v) coping and adaptation strategies, from which subthemes were developed within each category and illustrative quotations were selected to represent each subtheme. Participants were anonymised using identifiers such as “FGD-L1” (FGD from cluster 1) or “KII-L1.” The study methods and reporting were strengthened using elements of the COREQ (Consolidated Criteria for Reporting Qualitative Research) checklist to enhance transparency and reproducibility [34].

## Results

### Impacts of heat-related discomfort on health workforce

**Fig. 2:**
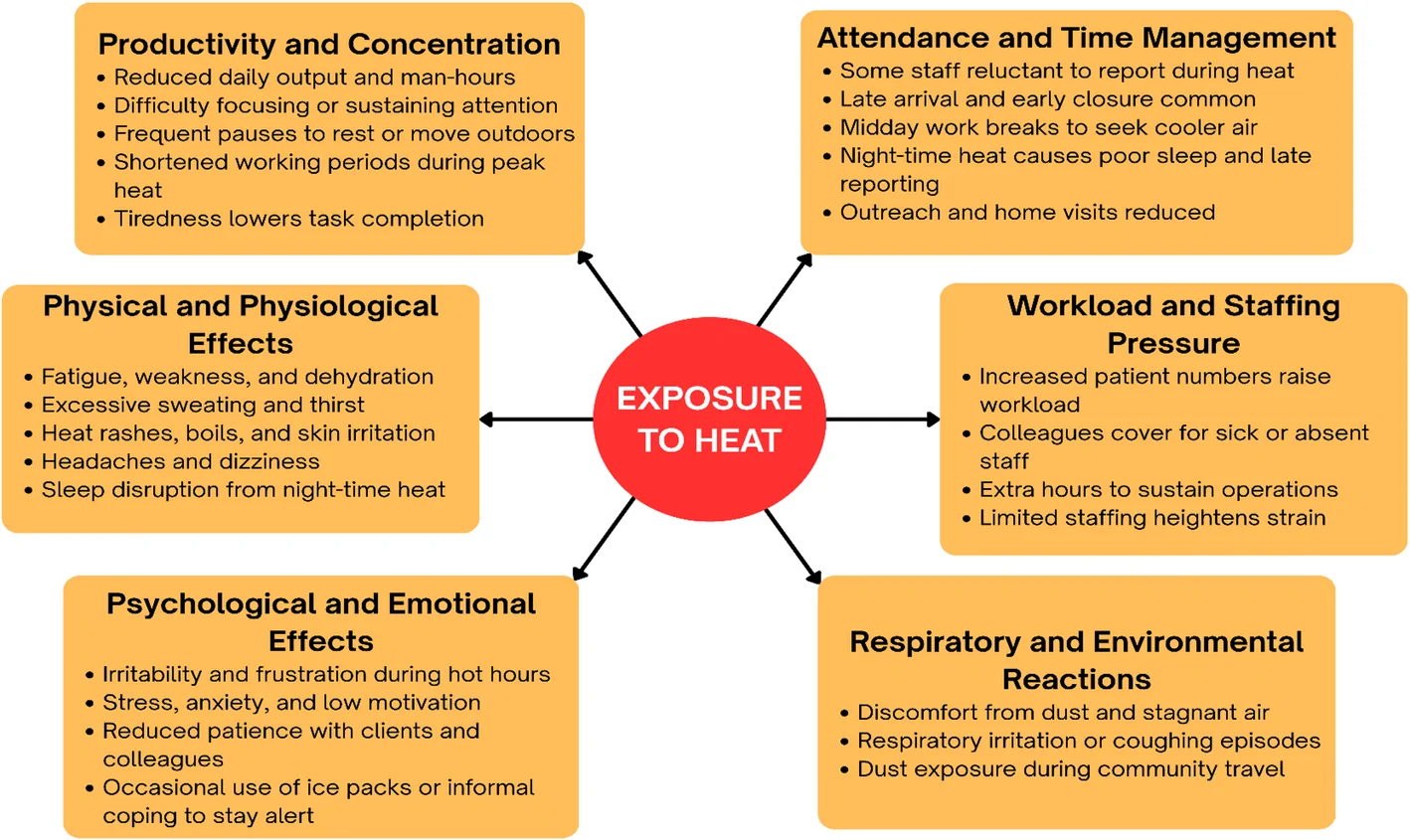
Reported impacts of heat-related discomfort on health workforce performance and wellbeing

Participant diversity captured multiple and differentiated perspectives from different levels of the health system. Across all regions and cadres, participants described a wide range of physical, psychological, and performance-related effects associated with heat-related discomfort in health facilities. These experiences clustered around six interconnected domains (Fig. 2): workload and staffing pressure, attendance and time management, productivity and concentration, physical and physiological effects, respiratory and environmental reactions, and psychological and emotional strain.

Increased workload and staffing pressure were widely reported during hot periods. Health workers described a surge in outpatient visits, which were often driven by dehydration, heat rashes, and respiratory complaints. One participant explained:

*“…there will be more case and there is tension at the OPD level…the way more people are falling sick and coming to the facility*,* so more workload on the health workers.”* (KII, L1)

Participants noted that these spikes in demand were particularly difficult in understaffed facilities, where remaining workers stepped in for colleagues who felt unwell or left early due to the heat. Exposure to heat also shaped attendance and time management. Participants reported late arrival, early closure, and reduced outreach activities on extremely hot days. Sleep disturbance caused by warm nights contributed to fatigue the following morning. As one health worker stated:

*“…if a staff is supposed to come to work at 8 o’clock… due to the [heat] stress*,* they end up coming to work a bit late*,* maybe an hour or two hours late.”* (KII, L1)

Participants perceived that these disruptions reduced the time available for patient care, as health workers consistently linked exposure to heat to lower productivity and concentration at work. They also described difficulties sustaining attention, frequent pauses to rest or move outdoors, and diminished task completion during peak heat hours:

*“You become so exhausted… you couldn’t sleep over the night… you can’t concentrate much… So the heat actually affects you and you are stressed as a health worker.”* (FGD, L4)

The cumulative effect was a reported slower work pace and reduced efficiency across departments by participants. Heat-related discomfort also produced several physical and physiological symptoms, as fatigue, excessive sweating, dizziness, headaches, and dehydration commonly mentioned. A participant noted:

*“There is heat stress and dehydration too… At times*,* the heat come to work and they are very weak.”* (KII, L3)

Lastly, participants highlighted significant psychological and emotional effects, including irritability, frustration, stress, and reduced patience with clients, with one who reflected as follows:

*“Looking at OPD… not all the fans are working…it will make them lose water…a little thing will trigger them to be angry and they will act towards their clients in an unfair manner.”* (KII, L3)

According to the participants, these emotional reactions often interacted with physical strain, in shaping overall performance and interpersonal dynamics as part of the impacts on the health workforce and related dynamics (Fig. 2).

### Impacts of heat-related discomfort on service delivery

**Fig. 3:**
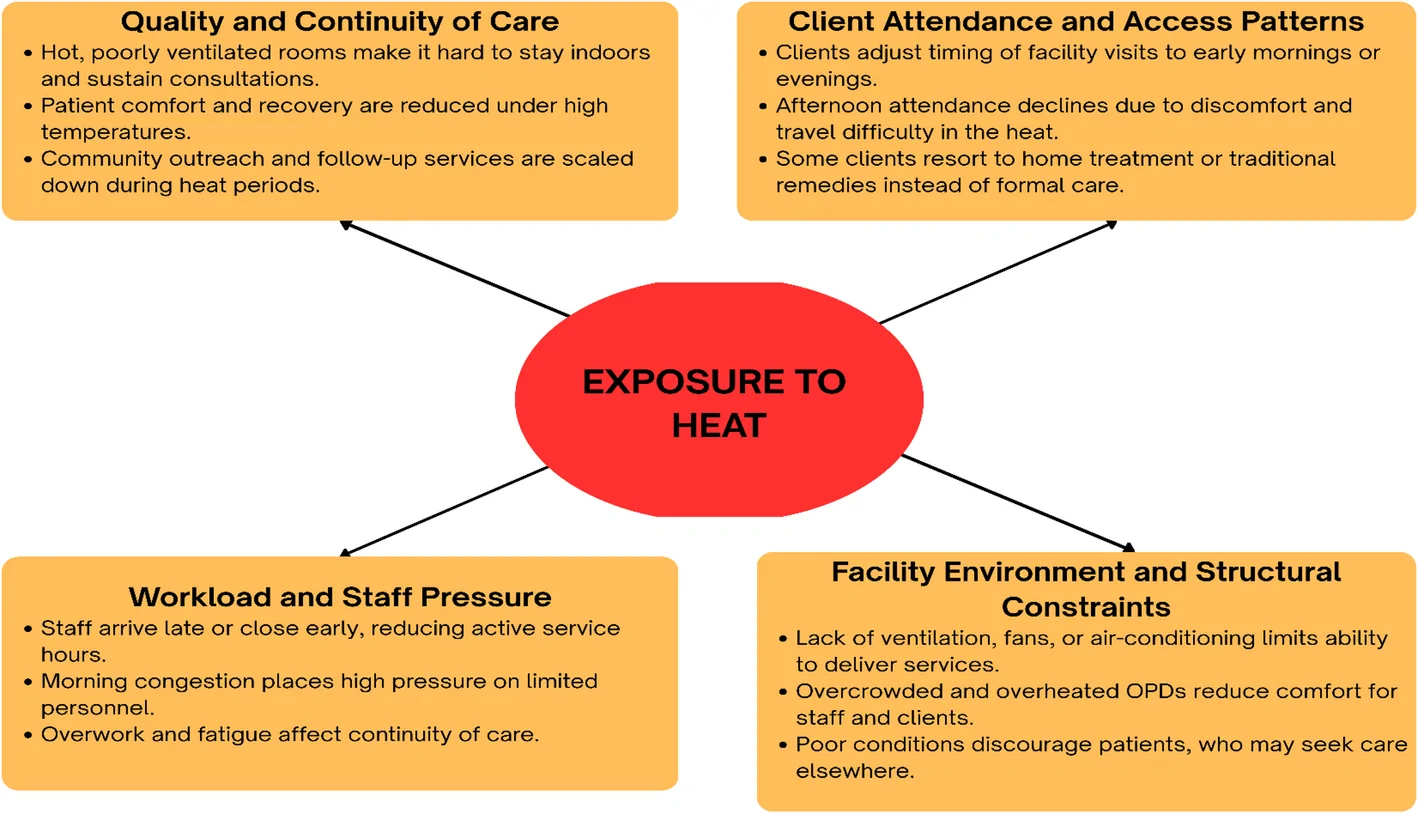
Reported impacts of heat-related discomfort on healthcare service delivery

Participants described multiple ways in which heat-related discomfort reshaped healthcare utilisation, constrained service delivery, and affected the quality and continuity of care. These impacts were closely linked to changes in client attendance patterns, staffing pressures, environmental conditions, and service delivery disruptions to clinical interactions (Fig. 3).

A notable theme concerned client attendance and access patterns, as participants reported that clients often adjusted their visiting times to early mornings or late evenings to avoid the afternoon heat, when both travelling to facilities and waiting indoors became difficult. One health worker explained:

*“…because of the extreme temperature*,* mostly in the afternoon*,* the clients will go in the morning… But if it reach afternoon*,* it becomes very difficult.”* (KII, L6)

Some clients reportedly chose to stay at home, self-medicate, or rely on traditional remedies with reasons of travel discomfort or waiting in overheated spaces. This reportedly contributed to irregular outpatient attendance and inconsistent service demand. Participants reported that poorly ventilated consulting rooms made it difficult to stay indoors, resulting in shortened consultations and reduced patient comfort, as noted:

*“When the heat becomes too much… patients lying in the ward…it can also prolong their recovery period… When health workers are stressed… to provide healthcare service will be impacted.”* (KII, L3)

Community outreach and patient/client follow-up services were also scaled down during periods of intense heat, affecting continuity of community-based health delivery. Workforce strain and staffing pressure reportedly emerged as recurring concerns of similar impacts. On the contrary, increased patient numbers during cooler periods heightened congestion and placed additional strain on the existing limited staff. A participant observed:

*“It becomes crowded… The stress… workload is high.”* (KII, L3)

Staff also arrived late or left early on extremely hot days, reducing active service hours and compressing workload into shorter time periods. These reportedly constrained staff capacity and reduced overall service provision. Participants emphasised that facility environment and structural constraints played a central role in shaping service quality. Lack of ventilation, fans, or air-conditioning, combined with overcrowded waiting areas made many indoor spaces uncomfortable for both staff and patients. One health worker described how this affected client retention:

*“When OPDs become choked… rooms become hot… patients may seek care elsewhere.”* (KII, L1)

Participants reported that some patients appeared to leave before being seen, which they attributed partly to heat-related discomfort, while others shifted to facilities perceived as cooler or better equipped. Environmental conditions reportedly intersect with staffing, infrastructure, and client behaviour to shape service performance across facilities (Fig. 3).

### Impacts of high temperature exposure on health infrastructure

**Fig. 4:**
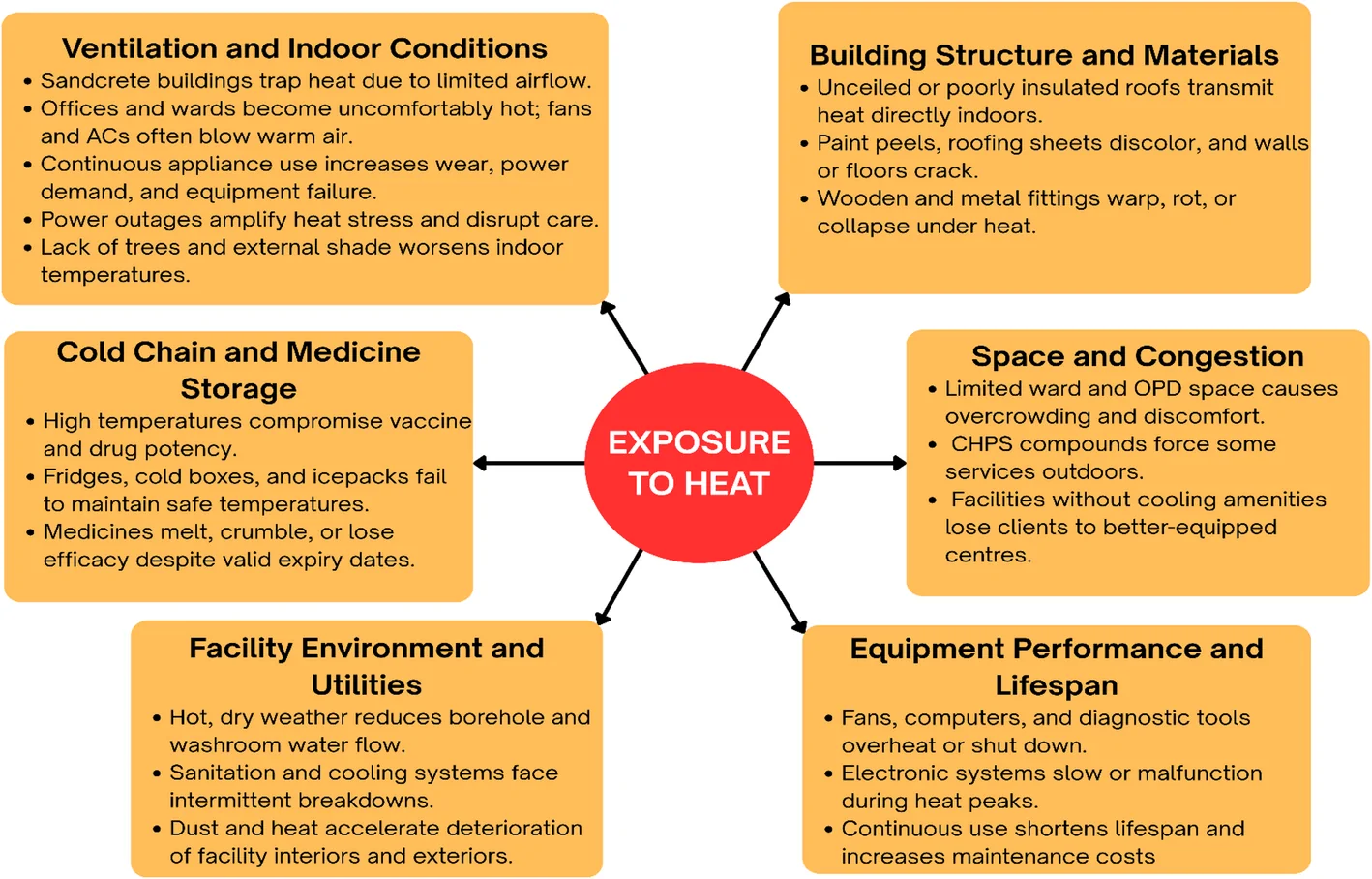
Reported impacts of high temperature exposure on health facility infrastructure

Participants described several ways in which high temperatures and inadequate heat regulation compromised the structural integrity, functionality, and operational capacity of health facilities. These effects (Fig. 4) were most pronounced in relation to ventilation and indoor conditions, building materials, cold chain and medicine storage, equipment performance, and utilities. A reported central concern was poor ventilation and indoor heat accumulation, particularly in modern sandcrete buildings with limited airflow. Participants explained that airtight windows, uninsulated/non-ceilinged roofs caused sharply higher daytime indoor temperatures, described by a participant:

*“With our sandcrete buildings… there’s no good ventilation. And when air is trapped in the heated area*,* the air also heats up*,* further compounding the issue.”* (KII, L6)

The continuous use of fans or air conditioners reportedly often provided limited relief, with appliances blowing warm air or failing under sustained thermal strain. Participants also noted that the absence of shade trees intensified indoor heat, as high temperatures were further linked to damage and deterioration of buildings and materials. Roofs discoloured, paint peeled, and cracks developed in walls and floors. Wooden doors and louvres warped or shrank in response to exposure to heat. As one participant observed:

*“…with this heat season…you see the buildings becoming dilapidated too early*,* the paint is peeling off…The doors and louvres…I don’t know whether to say bending or shrinking.”* (KII, L6)

Participants also highlighted important impacts on space and congestion. Small-sized wards and outpatient departments heated rapidly, causing discomfort for both patients and health workers. Some facilities were unable to accommodate large client volumes, particularly those lacking cooling systems, as was reported:

*“Once a lot of patients reported… we needed space to accommodate them… facilities were burdened with the heat because of the lack of space.”* (KII, L5)

Participants noted that in some communities, services had to be relocated outdoors or clients moved to better-ventilated centres. Exposure to heat also threatened cold chain integrity and medicine storage, particularly in facilities with unreliable power supplies. Participants described difficulties maintaining required temperatures for vaccines and heat-sensitive drugs as follows:

*“Most of our vaccines at times go to a certain degree that are not acceptable… when that happens*,* the potential [potency] of vaccines are always questionable… the heat passes through the roofing sheet… and affects most of the medicines.”* (KII, L6)

Participants reported concerns that medicines exposed to high temperatures could deteriorate physically and potentially compromise their quality. A related challenge concerned equipment performance and lifespan. Fans, computers, and diagnostic devices overheated and malfunctioned during extreme heat conditions. The continuous use to offset high indoor temperatures further shortened device lifespan and increased maintenance costs. A participant noted:

*“…because there is no ventilation… our laptops*,* computers… because of the weather*,* it will become hot and it easily breaks down.”* (KII, L6)

Lastly, participants described the broader effects of high temperatures on waterflow from boreholes as washrooms were abandoned during hot, dry periods, and pumps sometimes failed due to overheating, summed by the following observation:

*“For a district that depends very much on wells…sometimes you pump*,* and the flow is not that much”* (KII, L1)

### Governance and financing

Participants described a range of governance mechanisms (Box 1), that indirectly supported preparedness for high temperatures, although they emphasised that no dedicated heat-specific policy or guideline currently existed. Instead, existing institutional frameworks, including facility design standards, public health legislation, routine supervision, and emergency committees, provided partial support for managing exposure to heat within health facilities. Several respondents pointed to Ghana Health Service (GHS) infrastructure standards as generic fundamental governance tool. Uniform designs for CHPS and hospital structures were seen as helpful for promoting ventilation and functional space use as a participant explained:

*“If you are making policies… like the way Ghana Health Service has designed that all CHPS Zones are supposed to be built in a particular way… we have to make sure that those buildings… are able to sustain [the heat].”* (FGD, L1)

Others referred to national frameworks such as the Ghana Building Code and the Public Health Act (Box 1), indicating that these documents guided health facility construction and disaster preparedness, although implementation varied. As one respondent noted:

*“When you look at… the Public Health Act of 2012… in terms of disaster management… they have their policies in place.”* (KII, L1)

Internal oversight committees such as Public Health Emergency Committees and Risk Communication Teams were commonly cited as important platforms for coordinating education and contingency actions during environmental stress, even though there were notable gaps:

*“We have the emergency committee… but there are no specific plans. We only act when the contingencies come in.”* (KII, L2)

Participants described emerging local initiatives (i.e., tree planting, improved ventilation during renovations, and closer collaboration with planners and environmental health officers) as early signs of climate-sensitive governance (Box 1). Accordingly, some districts had begun discussing heat-related issues during planning meetings and performance reviews, though these were not yet formalised as policy.

Financing arrangements were seen as a major constraint, as participants explained that routine annual budgeting served as the primary entry point for any heat-related adaptation (Box 1), yet budgets contained no dedicated allocations for heat-related specific management or climate-health actions, noted in the following:

*“Everything starts with the budget. If you don’t add anything to your annual budget*,* then you find it difficult to do.”* (KII, L6)

Respondents emphasised the need for reliable funding sources, especially for maintaining cooling equipment, planting trees, or adding shade structures. In the absence of climate-specific budgets, facilities often relied on ad hoc partner support or community contributions. One participant stated:

*“There should also be a reliable funding source to tackle… in case of emergency… and the growing of trees needs to be intensified.”* (FGD, L6)

Healthcare workers reported that most heat-related actions required reallocating existing funds or using limited emergency provisions, which constrained the scale and sustainability of adaptation efforts. Collectively, these accounts indicate that governance frameworks currently provide indirect, fragmented support, while financing mechanisms remain insufficiently structured to address climate-related emergencies like the rising temperatures. Participants thought that resilience could be strengthened through explicit policy development, systematic integration of heat preparedness into routine planning cycles, and expanded budgetary support for climate-responsive facility improvements.

**Table:** 

Box 1. Governance and financing structures facilitating health system response to warm periods	
(a) Governance Mechanism	
• GHS design standards for facilities (uniform CHPS/hospital structures)	
• Ghana Building Code and Public Health Act references	
• Routine staff orientation and quarterly clean-ups	
• Risk communication and public health emergency committees	
• Network of Practice for referrals	
• Emerging climate-sensitive planning (tree planting, improved ventilation)	
(b) Financing Mechanism	
• Routine annual budgeting as entry point for adaptation	
• Integration of tree planting and ventilation improvements in budgets	
• Calls for reliable emergency funding sources	
• Ad hoc partner or community support for small-scale infrastructure	
• Limited but enabling managerial discretion in fund use	

### Coping and adaptation strategies

Participants listed multiple strategies used to sustain service delivery during periods of heat-related discomfort and exposure to high temperatures. These actions occurred at individual, facility, and system levels (Table 2).

At the individual level, coping responses were mainly behavioural and aimed at reducing personal heat strain. Health workers reported adjusting work hours to complete strenuous tasks before midday and pacing their activities during peak heat periods. They described hydrating frequently, taking brief breaks in shaded areas, and modifying clothing (such as wearing lighter uniforms or open footwear) to remain comfortable. Informal task rotation systems allowed colleagues to rest intermittently without interrupting service delivery. Participants also highlighted the role of self-motivation and peer support in maintaining focus and performance despite discomfort and fatigue. These practices were described as essential for sustaining basic functioning during the hottest parts of the day.

At the health facility level, centred on operational adjustments and modifications to the physical environment. Facilities attempted to improve ventilation by opening windows and doors, adding or repairing fans, and rearranging workspaces to enhance airflow. According to participants, when indoor conditions became intolerable, health workers temporarily relocated services such as OPD or immunisation sessions to shaded verandas or outdoor waiting areas. Rapid cooling measures, including applying wet towels or temporarily moving overheated patients outdoors, were used to prevent heat-related illness among both staff and clients. Facilities also reorganised workflows, assigning lighter duties to fatigued staff and triaging patients with severe conditions to reduce congestion. Adjustments to medicine storage and supply protocols were reportedly common, including placing heat-sensitive drugs in cooler rooms, adding ice packs to vaccine carriers, or more frequently monitoring refrigerator performance. Some facilities pooled internal resources to purchase fans or drinking water, while others-initiated tree planting or created shaded areas to reduce heat accumulation. These actions reflected improvised and nuanced context-specific responses intended to maintain service continuity.

At the system level, participants described adaptation as emerging through routine governance processes, capacity-building, and institutional learning. Orientation sessions increasingly incorporated occupational safety, hydration practices, and first aid for heat-related strain. Supervisory visits and routine review meetings considered environmental conditions and their implications for service delivery, particularly in relation to seasonal changes in patient load. Public Health Emergency Committees and Risk Communication Teams were activated during warmer periods to support local preparedness and disseminate information through community channels. Task redistribution and rotational staffing helped maintained service coverage when heat limited worker availability. The Ghana Health Service Network of Practice was reportedly used in some districts to refer patients when facilities became overcrowded or indoor spaces overheated, and few districts allocated contingency funds for maintaining cooling systems or incorporated tree planting into annual operational plans. Participants perceived that governance and planning structures were gradually becoming more responsive to environmental challenges although no formal policy explicitly addressed heat-related discomfort.

**Table 2:** Reported coping and adaptation strategies for managing heat-related discomfort and high temperature exposure in health facilities

Level	Category of strategy	Examples from data	Purpose/outcome
Individual (Coping)	Work hour and task adjustments	Starting shifts earlier; pacing work; avoiding fieldwork at midday	Reduce physical strain and exposure
	Hydration and rest	Carrying water; taking short breaks; resting in shaded areas	Prevent dehydration and fatigue
	Clothing modification	Wearing light uniforms, open shoes	Improve comfort and body cooling
	Informal rotation and self-motivation	Taking turns to rest while colleagues continue care; encouraging colleagues to persist	Maintain productivity, reduce exhaustion, and improve morale
Facility (Operational Adaptation)	Environmental management	Opening windows, adding fans, planting trees, creating shaded waiting areas	Improve ventilation and comfort
	Service reorganisation	Moving OPD or immunisation sessions outdoors; triaging severe cases	Maintain service flow and protect patients
	Rapid cooling and first aid	Applying wet towels, using fans, or moving overheated patients to cooler spaces	Prevent heat-related illness among patients and staff
	Equipment and medicine management	Monitoring refrigerators; adding ice packs; relocating heat-sensitive drugs	Preserve medicine potency and functionality
	Resource pooling	Collective purchase of fans and drinking water	Supplement limited institutional support
System (Institutional Adaptation)	Training and supervision	Incorporating heat and occupational safety into orientation and supervision	Build staff awareness and preventive skills
	Emergency preparedness and coordination	Public health emergency committees, risk communication teams	Facilitate timely planning and response
	Task and staffing management	Rotational leave planning, redistribution of duties	Ensure continuity of service under strain
	Referral and network systems	Using Network of Practice to decongest overcrowded facilities	Maintain patient care quality during peak heat
	Infrastructure and environmental planning	Tree-planting, contingency budgeting, minor facility retrofitting	Institutionalise resilience within health system planning

## Discussion

### Health workforce interpretation of health outcomes of heat-related discomfort

Drawing on qualitative evidence from health workers, the findings are broadly consistent with the guiding proposition that heat acts as a cross-cutting health system stressor affecting multiple WHO Health System Building Blocks simultaneously, rather than as an isolated occupational health concern, lessened through effective governance and financing mechanisms (Box 1). Thus, heat impacts supposedly cascade across interconnected domains of the health system, with effects in one area amplifying vulnerabilities in others, to reveal a systems-level vulnerability distinct from isolated occupational risks [8, 13, 21].

While existing research has documented exposure to heat effects on individual worker productivity and safety [5–8, 13, 14], the reported experiences from this study reveals that these individual-level effects have interconnected cascading effects. The documented clustering of physical, cognitive, and emotional effects potentially expands the occupational health literature in interconnectedness. Thus, physiological strain or sleep disturbance may reduce worker concentration, which may lead to procedural errors or irritability, which generates workplace stress, to further impair interpersonal relations with patients. Cognitive and slower performance among workers in West Africa and other parts of the world, particularly when cooling and hydration facilities are limited have been observed [10, 11, 19, 35–37]. In addition, physiological strain, sleep disturbance, and irritability reported in this study also reflect patterns identified in previous work on heat stress and mental fatigue among farmers and mining workers in Ethiopia and Ghana [36, 38]. Participants described patterns suggesting that heat-related challenges may cascade across interconnected domains, and highlight the vulnerability of health personnel where rising temperatures compound existing resource and infrastructure challenges.

The data also reveal that heat-driven behavioural adaptations by health workers and clients (such as taking frequent breaks, limiting movement and care-seeking to cooler parts in the day, or delaying tasks) occur simultaneously and interact in ways that amplify service delivery disruptions, an area less explored in existing studies [22, 35]. Previous studies have explored patient care-seeking behaviour during periods of extreme heat, documenting patterns such as delayed health-seeking and preference for self-treatment [39–41]. However, this study contributes to the understanding of health worker care provision during these hot conditions, as well as how both patient and health worker adaptations co-occur and create mismatches. In such situations, facilities have reportedly reduced staff capacity and focus, while patient volumes were perceived to concentrate during cooler hours, potentially creating operational pressures that may affect patient experience and service delivery [42, 43]. These interactions, less explored in existing literature [22, 43], further suggests that heat management interventions targeting only providers or only patients will be insufficient, as system resilience requires coordinated responses across both groups.

The infrastructure impacts documented reveal insights about the relationships between built environment and climate exposure. Evidence from the study suggests that certain design choices, particularly the shift to modern sandcrete buildings with limited airflow, increase indoor heat accumulation beyond outdoor levels. This finding contributes to emerging discussions on how building design may influence thermal conditions in healthcare facilities [44–46], by revealing an unintended consequence of health infrastructure modernisation in tropical contexts: newer facilities may be more structurally robust but thermally vulnerable. In addition, findings suggest a possible feedback mechanism whereby heat-related equipment failures may increase maintenance pressures and operational constraints, where high ambient temperatures accelerate equipment failure. This may increase maintenance costs and replacement frequency, which strains the already limited budgets, hence delaying repairs, to further compromise operational capacity. Although some reports by WHO [47, 48] have documented cold chain challenges that may result from climate exposure, the findings indicate a ‘compounding climate vulnerability’ where initial exposure to heat triggers cascading failures across infrastructure systems (cooling, power, water, equipment) that appears underrepresented in the literature reviewed for this study. Further research is needed to examine whether these vulnerabilities interact in additive or multiplicative ways [49].

The findings on governance also reveal that institutional structures that could support heat preparedness exist (infrastructure standards, emergency committees, supervision systems), yet no explicit policies address thermal management. The findings suggest that existing institutional structures may not yet be fully oriented toward heat-related risks. This finding highlights the need for depth and reorientation of climate-health policies [21, 50, 51] in order to leverage existing institutional platforms while adding climate-specific mandates. The financing constraints documented point to the invisibility of current adaptation costs. Participants reported that facilities incur costs associated with managing heat-related challenges (through increased energy use, accelerated equipment replacement, and informal resource mobilisation) but these expenditures remain embedded within general operating budgets, leading to climate financing receiving low priority. Revealing these hidden costs and making them explicit could strengthen advocacy for systematic adaptation financing [50, 52, 53].

The multi-level coping and adaptation strategies documented demonstrate considerable improvisation and institutional flexibility. However, as noted in other population groups in SSA [4, 7, 54], many of the strategies described appeared informal and reactive in nature. These findings correspond with organisational literature suggesting that adaptive capacity often develops through iterative, small-scale adjustments rather than large-scale transformation [55]. Nonetheless, the pattern of repeated personal resource investment (health workers purchasing fans, bringing drinking water), ad hoc facility modifications, and budget reallocation also represents unsustainable coping that risks workforce burnout and system fragmentation. That said, from a health system resilience perspective, these adaptations may be insufficient on their own to achieve broader transformative resilience objectives [56]. Achieving this would require clear pathways of understanding, technical interventions, and governing structures that make heat preparedness an explicit, funded health system function [57].

### Implications for healthcare facilities, health workers, and adaptation planning

The findings suggest that heat should increasingly be recognised as an occupational health and health system performance issue rather than solely an environmental concern. With the evidence from this study integrating heat risk management into occupational health policies could therefore contribute to protecting health workers while strengthening service continuity during periods of elevated temperatures. Such approaches are consistent with global recommendations that identify occupational exposure to heat as an emerging workplace health risk requiring preventive action through workplace adaptation, worker protection measures, and organisational preparedness [13, 21]. At the national level, climate-resilient health system planning could more explicitly incorporate thermal comfort considerations into health infrastructure standards, facility accreditation processes, and climate-health adaptation strategies. Existing design standards for health facilities may benefit from greater attention to passive cooling approaches, including building orientation, cross-ventilation, roof insulation, reflective roofing materials, shaded outdoor waiting areas, vegetation buffers, and appropriate window-to-room ratios. Such interventions have been shown to improve thermal conditions while reducing dependence on energy-intensive cooling systems, particularly in tropical and resource-constrained settings [23, 47]. The findings further suggest that future health facility investments should incorporate climate-informed infrastructure assessments to ensure that newly constructed facilities remain functional under projected increases in temperature and heatwave frequency.

The results also underscore the importance of strengthening occupational protection for health workers. Occupational health guidance could incorporate practical heat-management measures such as hydration protocols, access to safe drinking water, scheduled recovery breaks, task rotation during peak heat periods, flexible work scheduling, heat awareness training, and procedures for recognising and responding to heat-related illness among staff. Establishing heat-health protocols for healthcare workers may be particularly important in facilities where prolonged use of personal protective equipment, overcrowding, or inadequate ventilation further increases thermal strain [10, 22]. Integrating exposure to heat indicators into routine occupational risk assessments could support more systematic monitoring of workforce wellbeing and help reduce productivity losses, fatigue-related errors, and occupational health risks.

At the district and subnational levels, health management teams could integrate heat-related indicators into routine supervision, facility performance reviews, emergency preparedness exercises, and quality improvement programmes. Thus, incorporating heat considerations into annual operational planning may facilitate earlier identification of facility vulnerabilities, including risks to medicine storage, cold-chain integrity, equipment functionality, water availability, and staff welfare. District-level preparedness efforts could also benefit from stronger collaboration between health directorates, meteorological agencies, local government authorities, environmental health units, and disaster management institutions to support anticipatory planning and heat-health early warning systems [48, 58]. At the facility level, facility managers may also consider incorporating heat preparedness into routine risk assessments, business continuity planning, and occupational safety programmes. Although many of these measures are relatively low cost, participants noted that more substantial interventions, including retrofitting older facilities, upgrading electrical systems, improving water security, or installing sustainable cooling technologies, would require dedicated financing and long-term investment.

The findings further demonstrate that adaptation planning cannot be confined to the health sector alone. Effective management of heat-related risks within healthcare facilities will require intersectoral collaboration involving agencies responsible for energy, housing, infrastructure, environment, water resources, urban planning, and local government. Reliable electricity supply, climate-responsive building regulations, urban greening initiatives, and sustainable water systems all influence the capacity of health facilities to maintain safe and effective operations during periods of extreme heat. Consequently, climate adaptation planning should recognise healthcare facilities as critical infrastructure requiring priority protection within broader national climate resilience strategies [47, 59]. Finally, the widespread reliance on informal strategies to adapt to heat-related discomfort observed in this study suggests that health workers are already bearing significant adaptation burdens. Long-term resilience will therefore require dedicated financing mechanisms, climate-sensitive workforce protection policies, heat-informed infrastructure planning, and systematic incorporation of heat preparedness into health system governance. Such investments have the potential not only to protect health workers and patients but also to strengthen the reliability, safety, and sustainability of healthcare delivery under a warming climate [23, 60].

## Limitations

Relevant limitations were identified, and involved the reliance on self-reported experiences, which may be influenced by recall or perception bias. In addition, because the interviews involved health workers describing their own performance and facility conditions, social desirability bias may have influenced the responses despite assurances of confidentiality. To reduce this, multiple respondent groups and data sources (FGDs and KIIs) across geographically diverse clusters were triangulated, allowing convergence of themes and strengthening analytic credibility. Although participants were drawn from diverse administrative regions, the findings may not represent all health facilities in Ghana, particularly private or highly resourced settings. The inclusion of participants from multiple regions nonetheless enhanced the transferability of findings across comparable settings. Thus, transferability is most appropriate to public-sector primary and district-level facilities in similar tropical LMIC settings with limited cooling infrastructure and variable energy reliability. Finally, data collection occurred over a limited period, and seasonal variations in exposure to heat could influence responses. In spite of these limitations, the study offers important evidence to inform policies and interventions aimed at improving health system resilience in responding to the impacts of heat.

## Conclusion

This study explored how heat-related discomfort and high temperature exposure reportedly influence health workers, service delivery, and infrastructure within Ghana’s health system, and how existing structures facilitate coping and adaptation. The findings suggest that elevated indoor temperatures can disrupt work performance, patient flow, and medicine storage, while further straining infrastructure and limited available resources especially in rural districts of Ghana. Although health workers implemented various coping and adaptive measures, these remained largely informal and were not guided by formal policy frameworks. The study illustrates how participants perceived heat-related discomfort as affecting multiple interconnected health system components. Participants’ experiences indicate that improving indoor thermal conditions, ensuring reliable energy supply, and strengthening occupational health guidance could support safer and more comfortable work environments. Institutional efforts that integrate climate considerations into facility planning, staff training, and operational management may enhance preparedness over time. Future research could integrate qualitative findings with quantitative health system metrics to better understand long-term implications. Second, the use of spatial techniques will help better explain the contribution of climate hazards to the impacts, and overlaid on the measurable indicators should support the deployment of health early warning.

## Supplementary Information

Below is the link to the electronic supplementary material.

Supplementary Material 1.

Supplementary Material 2.

## Data Availability

The transcripts generated and/or analysed during the current study are not publicly available as they are property of the Standards for Official Statistics on Climate-Health Interactions Project, but are available from the corresponding author on reasonable request.
