# Supplementary material for: Health worker experience of heat-related discomfort, service delivery, and related coping and adaptation in Ghana
Source: Discov Public Health. 2026 Aug 25;23(1):1359. doi: 10.1186/s12982-026-02537-2 (PMC13506612; doi:10.1186/s12982-026-02537-2)
Supplement: Supplementary file 1 [file 12982_2026_2537_MOESM1_ESM.docx]

**Supplementary File 1: Thematic Interview and Focus Group Discussion Guide (Heat-Specific Domains)**

**Title**: Thematic Domains for Exploring Health Worker Experience of Heat-Related Discomfort, Service Delivery, and Related Coping and Adaptation in Ghana

This supplementary file outlines the thematic domains and analytical structure used to guide qualitative data collection and analysis on extreme heat exposure among health workers in Ghana. The domains were derived from the broader SOSCHI qualitative instruments, which were implemented as part of a national exercise conducted in collaboration with the Ghana Health Service (GHS). In line with institutional data governance requirements, verbatim interview and discussion questions are available upon request.

Only heat-relevant components of the broader tools informed this manuscript. Data were generated through Focus Group Discussions (FGDs) and Key Informant Interviews (KIIs) with health workers across Ghana.

Researchers interested in the full instruments may contact the authors directly, subject to institutional approval.

A. Contextual and Exposure Mapping *(Applied across FGDs and KIIs)*

These domains captured:

- Geographic and facility context (administrative location and role of participant)
- Duration of service within the facility
- Locally experienced climatic events over recent years
- Seasonal patterns and frequency of extreme heat exposure

B. Focus Group Discussion (FGD) Thematic Domains
   1. Health Workforce Experiences under Extreme Heat

- Explored perceptions and shared experiences related to:
- Physiological and psychosocial effects of heat on health workers
- Implications for daily routines, workload, and productivity
- Preparedness for heat-related emergencies
- Exposure to disaster risk management or climate–health training relevant to heat
  2. Service Delivery under Heat Stress Focused on:
- Frontline experiences of delivering care during periods of extreme heat
- Perceived disruptions to service continuity, quality, and efficiency
  3. Infrastructure, Equipment, and Health Information Systems Examined:
- Heat-related challenges affecting physical infrastructure and equipment
- Facility-level measures to reduce vulnerability and support system functioning during high temperatures
  4. Health Financing Constraints and Opportunities Addressed:
- Financial barriers limiting preparedness and response to heat risks
- Perceived pathways for sustainable financing of climate resilience and emergency preparedness at facility level
  5. Governance and Leadership Responses Covered:
- Awareness of policies addressing heat-related health risks
- Expectations regarding leadership roles, facility management, and governance structures in mitigating heat impacts

C. Key Informant Interview (KII) Thematic Domains
   1. System-Wide and Population Impacts of Extreme Heat Explored insights on:

- Differential impacts of heat on population groups, health workers, service delivery, and infrastructure
- Effects of heat on care-seeking behaviour and facility access
  2. Facility Capacity and Workload Dynamics Focused on:
- Facility responses to increased patient volumes during extreme heat
- Management of staff shortages, absenteeism, and workload pressures
  3. Coping, Adaptation, and Response Measures Examined:
- Facility-level strategies implemented before, during, and after extreme heat events
- Adaptive practices to sustain service delivery and protect staff wellbeing
  4. Policy and Strategic Frameworks Covered:
- Existing policies, plans, or strategies addressing heat-related risks
- Perceived gaps and opportunities for strengthening policy responses
