# Supplementary material for: Health worker experience of heat-related discomfort, service delivery, and related coping and adaptation in Ghana
Source: Discov Public Health. 2026 Aug 25;23(1):1359. doi: 10.1186/s12982-026-02537-2 (PMC13506612; doi:10.1186/s12982-026-02537-2)
Supplement: Supplementary file 2 [file 12982_2026_2537_MOESM2_ESM.docx]

**Supplementary file 2: Coding Tree Used in Thematic Analysis (Heat-Specific Extract)**

**Title**: Health Worker Experience of Heat-Related Discomfort, Service Delivery, and Related Coping and Adaptation in Ghana

| **Theme** | **Subtheme/Code** | **Description** |
| --- | --- | --- |
| Impacts of High Temperature Exposure on the Health Workforce | Workload and Staffing Pressure | Increased patient attendance, heavier workloads, worsening chronic conditions, longer queues, staff shortages due to discomfort, redistribution of tasks. |
|  | Attendance and Time Management | Late arrival, early departure, irregular attendance, sleep disturbances from night-time heat, rescheduled outreach activities. |
|  | Productivity and Concentration | Reduced concentration and task efficiency, slower work pace, difficulty sustaining attention during warm hours. |
|  | Physical and Physiological Strain | Fatigue, sweating, dehydration, dizziness, headaches, skin irritations such as rashes and boils. |
|  | Respiratory and Environmental Reactions | Respiratory irritation linked to heat, dust, and stagnant indoor air. |
|  | Psychological and Emotional Wellbeing | Irritability, frustration, mental exhaustion, reduced motivation, diminished attentiveness to patients. |
| Impacts of High Temperature Exposure on Service Delivery | Changes in Healthcare Utilisation Patterns | Early-morning and evening care-seeking, reduced midday attendance, increased reliance on self-medication or traditional remedies. |
|  | Service Delays and Operational Disruptions | Shortened service hours, congestion during busy periods, disrupted workflow, movement of services outdoors, slower operations in high heat. |
|  | Quality and Continuity of Care | Shorter and interrupted consultations, patient and staff discomfort indoors, postponed outreach activities, disrupted continuity of services. |
|  | Facility Environmental Conditions | Poor ventilation, faulty fans or ACs, overcrowded indoor spaces, patient preference for better-cooled facilities, indoor heat reducing perceived service quality. |
| Impacts of High Temperature Exposure on Health Infrastructure | Structural and Building Integrity | Heat transfer through poorly insulated buildings, peeling paint, fading roofing sheets, cracks in surfaces, warping wooden features, heat-trapping architecture. |
|  | Cooling and Energy Systems | Fans and ACs breaking down, continuous cooling demand, increased electricity use, unreliable power supply affecting cooling. |
|  | Space Limitations and Congestion | Overcrowded wards and OPDs, services moved outdoors, increased patient load at well-cooled facilities. |
|  | Medicine and Vaccine Storage | Cold chain failures, refrigerators unable to maintain temperature, melting ice packs, heat-compromised medicines. |
|  | Equipment and Utility Reliability | Overheating of computers and diagnostic tools, frequent device malfunctions, water supply interruptions due to pump failures. |
| Governance and Financing | Policy and Institutional Frameworks | Absence of heat-specific policies, reliance on general disaster or occupational health guidelines, uneven enforcement of standards. |
|  | Leadership and Management Practices | Facility-level planning, staff orientation on protocols, clean-up exercises, risk communication activities, committee coordination. |
|  | Emerging Local Initiatives | Tree planting, improved building design, involvement of planners and environmental officers, climate issues appearing in review meetings |
|  | Financing Mechanisms | No dedicated heat-preparedness budget, reliance on routine budget cycles, reallocation of existing funds, occasional external support. |
| Coping and Adaptation Strategies (Individual-Level Coping) | Work Hour and Task Adjustments | Starting work earlier, pacing tasks, avoiding midday fieldwork. |
|  | Hydration and Rest | Drinking water, taking brief shaded rests. |
|  | Clothing Modifications | Wearing lighter uniforms or open footwear |
|  | Informal Rotation and Self-Motivation | Taking turns to rest, peer encouragement, maintaining morale. |
| Coping and Adaptation Strategies (Facility-Level Operational Adaptation) | Environmental Management | Opening windows, adding fans, planting trees, creating shade, rearranging workspace for airflow. |
|  | Service Reorganisation | Moving OPD or immunisation outdoors, triaging severe cases to reduce congestion. |
|  | Rapid Cooling and First Aid | Applying wet towels, using fans, moving overheated patients/staff to cooler spaces. |
|  | Equipment and Medicine Management | Monitoring refrigerators, adding ice packs, relocating heat-sensitive medicines. |
|  | Resource Pooling | Staff contributing funds for drinking water. |
| Coping and Adaptation Strategies (System-Level Institutional Adaptation) | Training and Supervision | Integrating heat management into orientation, including Occupational Safety measures. |
|  | Emergency Preparedness and Coordination | Activities of Public Health Emergency Committees and Risk Communication Teams. |
|  | Task and Staffing Management | Rotational leave, duty redistribution, modified schedules during hot seasons |
|  | Referral and Network Systems | Using the GHS Network of Practice to manage overcrowding in overheated facilities. |
|  | Infrastructure and Environmental Planning | Tree-planting initiatives, contingency budgeting, minor retrofitting for ventilation. |
